# Perceptions of Undergraduate Community Medicine Students on the Digital Community Health Advocacy Model Program (D-CHAMP)

**DOI:** 10.7759/cureus.107625

**Published:** 2026-04-23

**Authors:** Sumit Jethani, Siddharth Sekhri, Anshumali G, Varun Garg, Karuna Ratwani, Jyoti Kasana, Vinay K Tiwari

**Affiliations:** 1 Community Medicine, North Delhi Municipal Corporation (DMC) Medical College and Hindu Rao Hospital, New Delhi, IND; 2 Anaesthesiology, North Delhi Municipal Corporation (DMC) Medical College and Hindu Rao Hospital, New Delhi, IND; 3 Forensic Medicine, North Delhi Municipal Corporation (DMC) Medical College and Hindu Rao Hospital, New Delhi, IND; 4 Obstetrics and Gynaecology, Indraprastha Apollo Hospital, New Delhi, IND; 5 Periodontology, North Delhi Municipal Corporation (DMC) Medical College and Hindu Rao Hospital, New Delhi, IND; 6 Surgery, North Delhi Municipal Corporation (DMC) Medical College and Hindu Rao Hospital, New Delhi, IND

**Keywords:** community medicine, digital health, digital health advocacy, health communication, medical education, undergraduate students

## Abstract

Background

Digital community health advocacy has become an essential component of contemporary public health. However, structured training in digital advocacy remains limited in undergraduate medical education. The Digital Community Health Advocacy Model/Program (D-CHAMP) was conceptualized as a curriculum innovation to strengthen digital advocacy competencies among undergraduate community medicine students. This study aimed to assess students’ awareness, attitudes, self-efficacy, perceived benefits and barriers, and implementation outcomes related to D-CHAMP.

Methodology

A cross-sectional, descriptive study was conducted from September to December 2025 among undergraduate medical students at North DMC Medical College, Delhi. Using universal sampling, 104 students who had completed their community medicine postings participated. Data were collected through a structured, pre-tested, self-administered online questionnaire assessing awareness, attitudes, self-efficacy, perceived benefits and barriers, and implementation outcomes (acceptability, feasibility, and appropriateness). Descriptive statistics, chi-square test, t-test, analysis of variance, and Pearson correlation were used for analysis, with p-values <0.05 considered statistically significant.

Results

Overall, 92.3% of the students correctly understood the concept of digital health advocacy. Overall attitudes were positive (mean composite score = 3.67 ± 1.06), with strong support for formal training. Self-efficacy was moderate (mean = 3.30 ± 0.76), indicating the need for skill development. Acceptability (mean = 3.62 ± 0.86), appropriateness (mean = 3.60 ± 0.85), and feasibility (mean = 3.46 ± 0.80) scores demonstrated favorable perceptions of D-CHAMP. Major perceived benefits included wide reach (76%), increased youth engagement (69%), and cost-effectiveness (65%). Key barriers included a lack of digital skills, low community digital literacy, and limited institutional support. Self-efficacy showed strong positive correlations with acceptability (r = 0.663), feasibility (r = 0.556), and appropriateness (r = 0.630; p < 0.001).

Conclusions

Undergraduate medical students demonstrated positive perceptions and readiness to engage in digital community health advocacy. D-CHAMP was perceived as acceptable, feasible, and relevant for integration into undergraduate community medicine curricula. Strengthening digital competencies and addressing skill and institutional barriers can facilitate effective implementation, preparing future physicians for public health advocacy in a digital era.

## Introduction

Health advocacy is a core competency of medical professionals and an important component of community medicine. Physicians play a key role in addressing social determinants of health, reducing health inequities, influencing public policy, and promoting community participation in health-related decision-making. Modern medical education, therefore, emphasizes the role of doctors not only as clinicians but also as advocates who engage with communities and health systems to improve population health outcomes [1].

Traditionally, health advocacy has relied on face-to-face communication, community meetings, and print media. However, the rapid expansion of digital technologies has transformed the landscape of health communication. Digital platforms such as social media, mobile applications, websites, and online campaigns now play an important role in disseminating health information, shaping public perceptions, and mobilizing communities [2,3]. These tools enable real-time communication, broader outreach, and interactive engagement with diverse populations.

Globally, digital health advocacy has shown considerable potential in promoting health awareness, improving health literacy, and supporting public health responses during emergencies [4]. During the COVID-19 pandemic, digital platforms were widely used for risk communication, myth-busting, and community engagement, highlighting their importance in contemporary public health practice [5]. Digital advocacy is also particularly useful in addressing sensitive health issues such as mental health, sexual and reproductive health, and infectious diseases, where accessibility and anonymity can encourage participation and reduce stigma [6]. Furthermore, digital inclusion has increasingly been recognized as a social determinant of health, influencing access to health information and services [7].

In India, community medicine plays a central role in undergraduate medical education by developing competencies related to epidemiology, health promotion, disease prevention, and community engagement. Undergraduate medical students are well-positioned to act as future health advocates [8]. However, teaching in community medicine has largely focused on conventional advocacy approaches, with limited structured training in digital health communication and advocacy [9].

In the post-pandemic era, the gap between rapidly evolving digital health ecosystems and existing medical curricula has become more evident. Although medical students frequently use digital platforms in their personal lives [3], their formal training in applying these tools for professional health advocacy remains limited. Studies among medical students in India have reported variability in awareness and preparedness related to digital health tools, highlighting the need for structured educational interventions [10,11].

To address this gap, the Digital Community Health Advocacy Model/Program (D-CHAMP) was conceptualized as a curriculum innovation aimed at sensitizing and training undergraduate community medicine students in digital advocacy. Understanding students’ perceptions toward such initiatives is essential for evaluating their feasibility and acceptability.

Therefore, the present study was conducted to assess undergraduate community medicine students’ perception, awareness, attitudes, and perceived benefits and barriers regarding D-CHAMP.

## Materials and methods

This study was conducted from September to December 2025 as a cross-sectional, descriptive study to assess the perceptions of undergraduate medical students who had completed their community medicine postings in the Department of Community Medicine, North DMC Medical College, Delhi, a tertiary care teaching institution catering to undergraduate medical education. The department routinely conducts community medicine postings and public health-oriented training for undergraduate students, making it a suitable setting for evaluating a curriculum innovation related to health advocacy.

Sample size and sampling

The sample size for the study was estimated using the single population proportion formula: $n = \frac{Z_{\alpha/2}^2 \, p (1 - p)}{d^2}$, where Z_α/2_​ = 1.96, corresponding to a 95% confidence level; p = 0.47, based on a previous Indian study by Garg et al. [11] assessing perceptions related to digital health concepts among undergraduate medical students; and d = 0.10, the allowable absolute error. Based on these parameters, the minimum required sample size was calculated to be approximately 96 students. During the data collection period, all eligible undergraduate medical students were invited to participate using a universal sampling approach. A total of 104 students provided complete and valid responses, all of which were included in the final analysis.

Inclusion and exclusion criteria

We included undergraduate medical students who had completed their community medicine postings in the Department of Community Medicine during the study period and who voluntarily agreed to participate and provided informed consent. We excluded incomplete or duplicate questionnaire responses.

Description of D-CHAMP

D-CHAMP was conceptualized as a structured framework to assess and promote digital health advocacy competencies among undergraduate medical students. In the present study, D-CHAMP was operationalized through a set of questionnaire domains evaluating key components of digital advocacy, including awareness, attitudes, self-efficacy, perceived benefits and barriers, and implementation outcomes such as acceptability, feasibility, and appropriateness. Students’ perceptions regarding these domains were assessed using Likert-scale-based items, and composite scores were derived to evaluate the overall perception of D-CHAMP as a potential curriculum innovation.

Study tool

Data were collected using a structured, pre-tested, self-administered questionnaire developed by the investigator after reviewing relevant literature and validated instruments [12]. The questionnaire was pilot tested among a small group of undergraduate medical students (10-15 students) who were not included in the final study sample to assess clarity, relevance, and comprehensibility of the items. Feedback obtained during the pilot testing was used to make necessary modifications to the questionnaire. Content validity of the questionnaire was assessed by experts from the Department of Community Medicine and medical education, who reviewed the items for relevance and adequacy in measuring perceptions of digital health advocacy. The internal consistency of the Likert-scale items was evaluated using Cronbach’s alpha, which showed acceptable reliability (Cronbach’s alpha = 0.82), indicating good internal consistency of the instrument.

The questionnaire was administered using Google Forms and consisted of five sections, including sociodemographic details, awareness and attitudes toward digital health advocacy, and self-efficacy in digital health advocacy, which was assessed using adapted items from the General Self-Efficacy Scale. Responses were recorded on a five-point Likert scale ranging from 1 (“Not at all true”) to 5 (“Exactly true”). Other sections included perceived benefits and barriers to digital advocacy, which were evaluated using items adapted from the Technology Acceptance Model and the Digital Health Literacy Instrument framework. Feasibility and acceptability of D-CHAMP were measured using validated items adapted from the Acceptability of Intervention Measure and Feasibility of Intervention Measure, rated on a five-point Likert scale ranging from “Strongly disagree” to “Strongly agree” [12]. Internal consistency of Likert-scale items was assessed, and the finalized questionnaire was used for data collection. Normality of composite scores was assessed using graphical methods (histogram/Q-Q plot) and found to be approximately normal.

Data management and analysis

Data were exported from Google Forms to Microsoft Excel (Microsoft Corp., Redmond, WA, USA) and analyzed using SPSS version 25 (IBM Corp., Armonk, NY, USA). Descriptive statistics included frequencies and percentages for categorical variables and mean with standard deviation (SD) for continuous variables and Likert-scale scores. Inferential analysis was performed using the chi-square test or Fisher’s exact test for categorical variables and the independent t-test or one-way analysis of variance for comparison of mean scores between groups. A p-value <0.05 was considered statistically significant.

## Results

A total of 104 undergraduate medical students participated in the study. The mean age of the participants was 20.96 years (SD = 1.23), ranging from 19 to 25 years. Of the respondents, 67 (64.4%) were male, and 37 (35.6%) were female.

Regarding conceptual understanding, 96 (92.3%) respondents correctly identified digital health advocacy as promoting health using digital platforms. A small proportion, 6 (5.8%), interpreted it as providing online treatment, and 1 (1.0%) selected none of the above.

Students identified several digital platforms suitable for community health advocacy, and multiple responses were allowed. WhatsApp was selected by 65 (62.5%) respondents, followed by Facebook by 57 (54.8%), and mobile applications by 57 (54.8%) respondents.

Students demonstrated generally positive attitudes toward digital community health advocacy (Table 1). The statement “Digital advocacy is effective for community health” had the highest mean score (3.82 ± 1.25). Other items included “Digital platforms can reach wider audiences” (mean = 3.63 ± 1.13), “Medical students should be trained in digital advocacy” (mean = 3.59 ± 1.08), and “D-CHAMP can improve my advocacy skills” (mean = 3.66 ± 1.20). The overall composite attitude score was 3.67 ± 1.06.

**Table 1:** Attitudes toward digital community health advocacy among students. The data is represented as mean ± SD. D-CHAMP = Digital Community Health Advocacy Model/Program; SD = standard deviation

Attitude item	Mean	SD
Digital advocacy is effective for community health	3.82	1.25
Digital platforms can reach wider audiences	3.63	1.13
Medical students should be trained in digital advocacy	3.59	1.08
D-CHAMP can improve my advocacy skills	3.66	1.20
Composite attitude score	3.67	1.06

Self-efficacy scores in digital health advocacy ranged from 3.20 to 3.47 across individual items, with an overall composite self-efficacy score of 3.30 ± 0.76. For implementation outcomes (Table 2), acceptability scores ranged from 3.57 to 3.67, with a composite mean of 3.62 ± 0.86. Appropriateness scores ranged from 3.59 to 3.61, with a composite mean of 3.60 ± 0.85. Feasibility scores ranged from 3.34 to 3.63, with a composite mean of 3.46 ± 0.80.

**Table 2:** Acceptability, feasibility, and appropriateness scores of the D-CHAMP curriculum innovation. The data is represented as mean ± SD. D-CHAMP = Digital Community Health Advocacy Model/Program; AIM = Acceptability of Intervention Measure; FIM = Feasibility of Intervention Measure; IAM = Intervention Appropriateness Measure; SD = standard deviation

Implementation outcome	Item mean range	Composite score (mean ± SD)
Acceptability (AIM)	3.57–3.67	3.62 ± 0.86
Feasibility (FIM)	3.34–3.63	3.46 ± 0.80
Appropriateness (IAM)	3.59–3.61	3.60 ± 0.85

The most commonly reported perceived benefit of digital community health advocacy was wide reach (79, 76%), followed by increased awareness among youth (72, 69%) and cost-effectiveness (68, 65%). Real-time engagement with communities was reported by 44 (42%) respondents.

**Figure 1:**
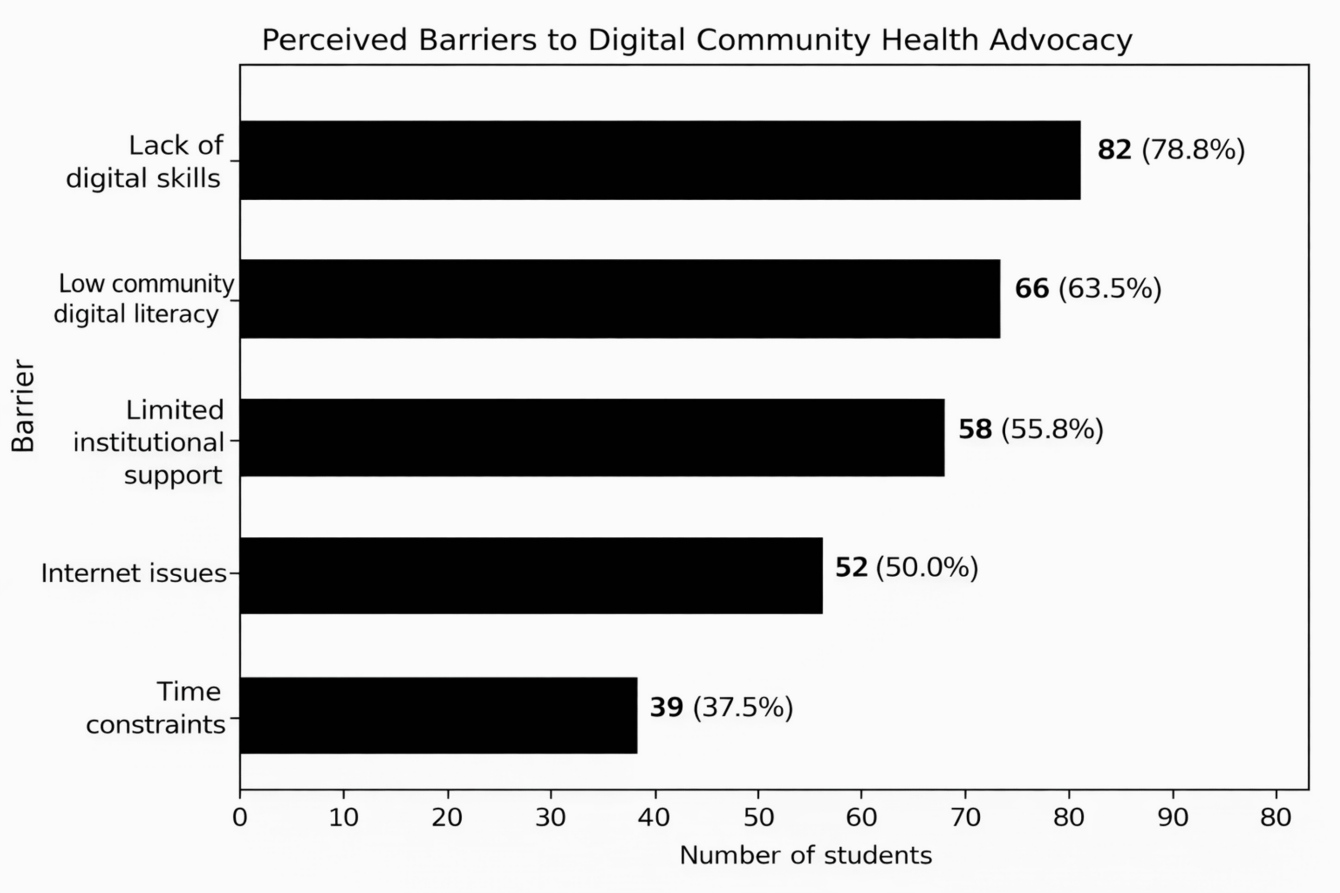
Perceived barriers to digital community health advocacy. The data is represented as N (%).

Participants identified several barriers to digital community health advocacy, including a lack of digital skills, low community digital literacy, limited institutional support, internet connectivity issues, and time constraints (Figure 1).

Inferential statistical analysis showed no significant association between gender and awareness of digital health advocacy in the chi-square test (χ² = 0.021, p = 0.884). An independent t-test comparing attitude scores by posting completion was not statistically significant (t = −1.259, p = 0.211). Similarly, the independent t-test showed no significant difference in attitude scores by gender (t = −1.259, p = 0.211).

However, Pearson correlation analysis (Table 3) demonstrated significant strong positive correlation between self-efficacy and implementation outcomes, including acceptability (r = 0.663, p < 0.001), feasibility (r = 0.556, p < 0.001), and appropriateness (r = 0.630, p < 0.001).

**Table 3:** Correlation between self-efficacy and implementation outcomes. P-values <0.05 are considered significant. AIM = Acceptability of Intervention Measure; FIM = Feasibility of Intervention Measure; IAM = Intervention Appropriateness Measure; SD = standard deviation

Variables	Mean ± SD	Correlation coefficient (r)	P-value
Self-efficacy	3.30 ± 0.76	-	-
Acceptability (AIM)	3.62 ± 0.86	0.663	<0.001
Feasibility (FIM)	3.46 ± 0.80	0.556	<0.001
Appropriateness (IAM)	3.60 ± 0.85	0.630	<0.001

## Discussion

The present study assessed undergraduate community medicine students’ perceptions of D-CHAMP in terms of awareness, attitudes, self-efficacy, perceived benefits and barriers, and implementation outcomes such as acceptability, feasibility, and appropriateness. The findings provide insight into the readiness of medical students to engage with digital advocacy as an emerging competency within community medicine education.

More than 90% of participants correctly identified digital health advocacy as the use of digital platforms for health promotion. Similar observations have been reported in previous studies, suggesting that medical students often develop familiarity with digital health concepts through routine engagement with social media and online platforms rather than through formal curricular training [2,13]. While this informal exposure contributes to conceptual understanding, structured academic integration is necessary to translate this familiarity into professional competence.

Students demonstrated generally positive attitudes toward digital community health advocacy, with the composite attitude score exceeding the neutral midpoint of the Likert scale. Participants agreed that digital platforms are effective tools for community health promotion and should be incorporated into undergraduate medical training. These findings are consistent with earlier studies reporting favorable perceptions of digital tools and social media for health communication among healthcare professionals [2-4,13]. The increased reliance on digital communication during the COVID-19 pandemic has further highlighted the importance of digital advocacy in public health practice [5,10]. Notably, attitude scores did not differ significantly across gender or academic exposure variables, suggesting broad acceptance of digital advocacy among students.

Despite positive attitudes, students reported only moderate levels of self-efficacy related to digital advocacy. While they were comfortable using digital platforms, many lacked confidence in managing challenges, sustaining advocacy initiatives, or creating impactful digital health content. Similar gaps between perceived importance and practical confidence have been reported in studies assessing digital health and telemedicine preparedness among medical students [10,11]. These findings emphasize the need for structured training, practical exercises, and mentorship within the D-CHAMP curriculum to enhance students’ digital advocacy skills.

Students rated D-CHAMP as highly acceptable and appropriate, indicating strong support for integrating digital advocacy training into community medicine education. The high appropriateness scores reflect the alignment between digital advocacy and the core objectives of community medicine, including health promotion, community engagement, and addressing social determinants of health [1,8]. Feasibility scores, although positive, were slightly lower, suggesting potential implementation challenges such as limited curricular time, variable institutional support, and infrastructural constraints, which have also been reported in Indian medical education settings [7,9].

Participants identified wide reach, increased youth engagement, and cost-effectiveness as major benefits of digital advocacy. These perceptions are consistent with global evidence demonstrating the ability of digital platforms to disseminate health information rapidly and engage younger populations [2,4]. Real-time engagement was also recognized as an advantage, particularly during public health emergencies when timely communication is essential [5,6].

The most commonly reported barrier was a lack of digital skills, followed by low community digital literacy and limited institutional support. These challenges are widely documented in digital health literature, particularly in low- and middle-income countries where digital divides persist [7]. Addressing these barriers will require a multi-level approach involving student training, faculty development, supportive institutional policies, and improved digital infrastructure.

A notable finding of the study was the strong positive correlation between self-efficacy and implementation outcomes, namely, acceptability, feasibility, and appropriateness. Students with higher confidence in their digital skills were more likely to perceive D-CHAMP as feasible and relevant. This aligns with educational research highlighting self-efficacy as a key determinant of engagement with new learning interventions [1,13].

Overall, the findings support the integration of digital community health advocacy into undergraduate community medicine curricula. Digital advocacy complements competency-based medical education goals related to communication, leadership, professionalism, and health promotion, and may help align traditional community medicine training with the evolving landscape of contemporary public health practice.

Strengths and limitations

The study’s strengths include the use of validated instruments, assessment of multiple implementation outcomes, and inclusion of inferential analyses to identify key determinants of acceptance. However, the study was conducted in a single institution, which may limit generalizability. Additionally, reliance on self-reported data may introduce social desirability bias. Future multi-centric and longitudinal studies are recommended to evaluate the long-term impact of digital advocacy training on student behavior and community health outcomes.

## Conclusions

The present study assessed awareness, attitudes, self-efficacy, and implementation outcomes related to D-CHAMP among undergraduate community medicine students. Although awareness of digital health advocacy was limited, students demonstrated adequate conceptual understanding of its role in public health. Overall attitudes toward digital advocacy were positive, with strong support for incorporating structured digital advocacy training into the undergraduate curriculum. The high acceptability, appropriateness, and feasibility scores indicate that D-CHAMP is perceived as a relevant and implementable educational innovation in community medicine. However, students reported moderate levels of self-efficacy, highlighting a gap between theoretical understanding and practical competence. Key perceived benefits included wider reach, improved youth engagement, and cost-effectiveness, while major barriers identified were limited digital skills, low community digital literacy, and insufficient institutional support. In conclusion, while undergraduate students show readiness and favorable perceptions toward digital community health advocacy, strengthening digital competencies through structured, skill-based training is essential. Integrating D-CHAMP into the undergraduate curriculum may enhance students’ capacity for effective public health advocacy in the evolving digital landscape.

## Appendices

**Table 4:** Study questionnaire.

Section	Q. No.	Question
Section A: Demographic information	1	Age: __________
	2	Gender: (a) Male (b) Female (c) Prefer not to say
	3	Year of study: 3rd-year MBBS
	4	Have you completed your Community Medicine posting? (a) Yes (b) No
Section B: Awareness about digital health advocacy	5	Have you heard the term Digital Health Advocacy before today? (a) Yes (b) No
	6	Which of the following best describes Digital Health Advocacy? - Promoting health through digital platforms (social media, apps, websites) - Providing online treatment to patients - Developing hospital software - None of the above
	7	Which platforms can be used for digital community health advocacy? (Select all that apply) Facebook, Instagram, WhatsApp, YouTube, Twitter/X, Blogs, Mobile apps, Other
Section C: Attitudes toward digital advocacy (Scale: 1 = strongly disagree → 5 = strongly agree)	8	Digital advocacy is an effective tool for promoting community health
	9	Digital platforms can reach a wider audience than traditional health education
	10	Community medicine students should be trained in digital advocacy
	11	I believe D-CHAMP can improve my advocacy skills as a future physician
	12	Digital advocacy is relevant to the current healthcare scenario
Section D: Self-confidence in digital advocacy (Adapted from GSES) (Scale: 1 = Not at all true | 2 = Hardly true | 3 = Moderately true | 4 = Mostly true | 5 = Exactly true)	13	I can always manage to solve problems if I face difficulties while creating digital health advocacy content
	14	If someone opposes my digital health advocacy ideas, I can find ways to get my message across
	15	I am confident that I can use digital tools (social media, blogs, apps) effectively for advocacy
	16	It is easy for me to stick to my digital advocacy goals and accomplish them
	17	I can remain calm when facing technical challenges because I can rely on my coping abilities
	18	When I am confronted with a problem in digital advocacy, I can usually find several solutions
	19	If I am in trouble while working on digital advocacy, I can usually think of something to do
	20	No matter what difficulties I face, I can usually handle them if I am engaged in digital advocacy tasks
	21	If I invest the necessary effort, I can successfully carry out digital health advocacy
	22	I can handle unexpected situations related to digital advocacy effectively
Section E: Benefits and barriers	23	What do you think are the benefits of digital advocacy? (Select all that apply) Wide reach, Cost-effective, Real-time engagement, Increases awareness among youth, Other
	24	What do you think are the barriers to digital advocacy? (Select all that apply) - Lack of digital skills, Limited internet access, Lack of institutional support, Time constraints, Low community digital literacy, Other
Section F: Feasibility and acceptability of D-CHAMP (Adapted from AIM/FIM/IAM) (Each domain has 4 items. Use a 5-point Likert scale: 1 = Strongly Disagree | 2 = Disagree | 3 = Neutral | 4 = Agree | 5 = Strongly Agree)	25	I welcome digital health advocacy training as part of medical education
	26	Digital health advocacy training is appealing to me
	27	I like the idea of digital advocacy training for undergraduate students
	28	Digital health advocacy training meets my expectations as a learner
	29	Digital health advocacy training seems implementable in my institution
	30	Digital advocacy training would be easy to carry out in practice
	31	I believe students can easily engage with digital advocacy activities
	32	Digital health advocacy training would be possible to introduce within the current curriculum
	33	Digital health advocacy training seems like a good fit for undergraduate community medicine
	34	Digital advocacy training is suitable for preparing us as future doctors
	35	Digital health advocacy training is relevant to the goals of medical education
	36	This type of training makes sense for undergraduate students
